# Validation of the patient-reported experience measure for care in Chinese hospitals (PREM-CCH)

**DOI:** 10.1186/s12939-020-01370-6

**Published:** 2021-01-07

**Authors:** Xuanxuan Wang, Jiaying Chen, Yaling Yang, Bo Burström, Kristina Burström

**Affiliations:** 1 School of Health Policy and Management, Nanjing Medical University, No. 101 Longmian Avenue, Nanjing, 211166 Jiangsu Province China; 2 Institute of Healthy Jiangsu Development, Nanjing Medical University, No. 101 Longmian Avenue, Nanjing, 211166 Jiangsu Province China; 3 Centre for Health Policy Studies, Nanjing Medical University, No. 101 Longmian Avenue, Nanjing, 211166 Jiangsu Province China; 4 Creative Health Policy Research Group, Nanjing Medical University, No. 101 Longmian Avenue, Nanjing, 211166 Jiangsu Province China; 5 Nuffield Department of Primary Care Health Sciences, Medical Sciences Division, University of Oxford, New Radcliffe House, Walton Street, Oxford, OX2 6NW UK; 6 Equity and Health Policy Research Group, Department of Global Public Health, Karolinska Institutet, 171-77 Stockholm, Sweden; 7 Health Outcomes and Economic Evaluation Research Group, Stockholm Centre for Healthcare Ethics, Department of Learning, Informatics, Management and Ethics, Karolinska Institutet, 171-77 Stockholm, Sweden

**Keywords:** Patient experience, Patient-reported experience measure, Psychometric evaluation, Public hospital, China

## Abstract

**Background:**

A psychometrically validated instrument to measure patient experience in Chinese public hospitals would be useful and is currently lacking. Our research team developed the Patient-Reported Experience Measure for Care in Chinese Hospitals (PREM-CCH). We aimed to validate this PREM-CCH in the present study.

**Methods:**

Data were drawn from a cross-sectional patient survey in 2016. Complete responses from 2293 outpatients and 1510 inpatients were included. Separate psychometric evaluation was carried out on outpatient and inpatient PREM-CCHs in terms of exploratory factor analysis, internal consistency, construct validity and criterion validity.

**Results:**

The validated outpatient PREM-CCH contained 22 items and five Factors, i.e. *Communication and information*, *Professional competence*, *Medical costs*, *Efficiency*, and *Hospital recommendation*. The validated inpatient PREM-CCH contained 19 items and six Factors, i.e. *Communication and information*, *Professional competence*, *Medical costs*, *Efficiency*, *Health outcomes*, and *Hospital recommendation*. The PREM-CCH showed satisfactory internal consistency, construct validity and criterion validity.

**Conclusions:**

The PREM-CCH is one of the first validated instruments capturing patient experience of care in the context of Chinese public hospitals. It performed well in the psychometric evaluation. It consists of a basic set of items important to patients that could be applicable to public hospitals in China and actionable to inform quality improvement initiatives.

**Supplementary Information:**

The online version contains supplementary material available at 10.1186/s12939-020-01370-6.

## Introduction

China has a hospital-centered health-care delivery system. Public hospitals consume a huge amount of health resources of both outpatient and inpatient care in China [1]. In 2019, Chinese public hospitals provided 37.5% of the outpatient visits and 65.8% of the inpatient admissions, and accounted for almost half of the total health expenditure (48.0%) [2]. As one core area of China’s new round of health-care system reform, pilot reforms for public hospitals covering both urban and rural areas were initiated in 2010, aiming to control the escalating growth of health expenditure and improve the quality and efficiency of care provided in public hospitals [3]. The achievement of providing affordable and equitable basic health care for all, the goals of China’s health-care system reform, is largely depending on the success of the public hospital reform [4]. Many studies have been conducted to evaluate the public hospital reform from different aspects [5–8]. However, the patient perspective, has rarely been considered in previous studies.

Studies have shown that high ratings based on patient experience are correlated with higher clinical quality [9]; therefore, patient experience can be a critical component of quality of care and a basis to develop actionable plans to improve the performance of health-care providers [10]. As survey instruments to capture patient experience, Patient-Reported Experience Measures (PREMs) can elicit feedback from patients regarding whether certain factors important to patients occur or not [11]. PREMs can be used complementarily to Patient-Reported Outcome Measures (PROMs) to give an overall assessment of quality of care [12–15]. Patient experience has been highlighted at the national level in many European countries and patient experience data have been increasingly used worldwide to evaluate the performance of health-care providers and to stimulate quality improvement [16–20].

Along with the deepening of China’s health-care system reform, patient-centeredness has been increasingly recognized as a critical concept to be included in reform policies to adopt the perspective and need of the patient [21]. A growing number of studies have been conducted by Chinese scholars to explore how to use PREMs to improve quality of hospital care [22, 23]. However, previous studies focus solely on outpatient services or inpatient care, and were conducted either in urban areas or in rural areas [24–26]. The only instrument used to collect information on both outpatient and inpatient experience across the country was developed based on published questionnaires rather than priorities listed by patients in a Chinese setting, i.e. did not adopt a patient perspective [27]. The sampled hospitals in that study were all located in urban areas. Although it was mentioned that psychometric evaluation was carried out for that instrument, only internal consistency was assessed with Cronbach’s α as proof [28]. A thorough psychometrically validated instrument to measure patient experience in public hospital settings in China would be useful and is currently lacking.

Our research team has developed an instrument to measure patient experience of care in the context of public hospitals in China covering both urban and rural areas, i.e. the Patient-Reported Experience Measure for Care in Chinese Hospitals (PREM-CCH), and the development process has been reported elsewhere [29]. The PREM-CCH was a survey instrument containing 42 items on patient experience. In the present study, we aimed to validate this PREM-CCH in measuring patient experience of care provided in Chinese public hospitals. The validated instrument could be used in the performance evaluation of public hospitals. Both health authorities and hospital leaders could take advantages of using the PREM-CCH, as the evaluation outcomes could inform future quality improvement practice that take priority from the patient’s perspective.

## Methods

### Study design

Data were drawn from a cross-sectional patient survey conducted by our research team in 2016, where the PREM-CCH was used as the patient experience survey instrument. Details on the sampling principle of participating hospitals and patients were reported elsewhere [29]. A total of 9 city-level (in the urban area) and 16 county-level (in the rural area) public hospitals were sampled through consultations with officials from China’s National Health and Family Planning Commission (NHFPC), the former counterpart of China’s National Health Commission, and stratified sampling. On the first stage, by consulting with the officials from the System Reform Division of NHFPC, our research team selected 5 cities and 8 counties from 4 provinces covering the eastern, central and western China where tangible reforms on public hospitals were implemented. On the second stage, according to the economic development and population size, in the same provinces our research team selected another 4 cities and 8 counties where public hospital reforms were not initiated in 2016. On the third stage, the largest municipal general hospital in each sampled city and the largest county-level general hospital in each sampled county were selected as sampled hospitals. A convenience sample of 200 outpatients and 100 inpatients in each sampled hospital was planned to be obtained. The inclusion criteria were that the participating outpatients should have completed their visits and the participating inpatients would be discharged soon. The patients who could not answer the questions themselves were excluded from the survey. Altogether 4951 outpatients and 2605 inpatients participated in the survey and responded to the PREM-CCH.

### The PREM-CCH instrument

The PREM-CCH instrument was development through a multi-stage approach. Firstly, a qualitative study was conducted to elicit what patients cared about most when seeking care in Chinese public hospitals in urban and rural areas [30]. The priorities determined was exclusively from the perspectives of patients rather than medical professionals or health authorities. Based on that qualitative study, an extensive literature review was performed to identify relevant items in validated PREMs. Afterwards, comparisons were made between results from the qualitative study and literature review to select items that were prioritized by patients receiving care in Chinese public hospitals and could be measured by validated instruments available. Finally, a pilot study was conducted to examine readability and comprehension of the instrument. Appropriate changes were then made accordingly. The final version of the PREM-CCH instrument consisted of 42 items on patient experience and one item on the general satisfaction with hospital care. Two patient experience items were exclusive to inpatients, while the others were applied to both outpatients and inpatients.

### Ethical consideration

In accordance with *The Ethical Review Policy of Human Biomedical Research* issued by China’s former Ministry of Health, ethics approval was unnecessary for our patient survey, because only patient interviews were conducted without any physical, chemical or biological approach [31]. Nevertheless, our research team did follow standard procedures to obtain oral informed consent from each participant before the interview started [29].

### Data cleaning

Of the 4951 participating outpatients, 4835 had no missing data in general characteristics and 3986 were 18 years old and above. Among the 40 items on outpatient experience, two items were excluded due to the high rates of “not applicable” responses (≧30%) [32]. As in the present study, exploratory factor analysis was one principal method, which cannot be performed if there are missing data, we excluded cases with responses “cannot tell” (*n*=350) and “not applicable” (*n*=1343) for patient experience items. Ultimately, 2293 cases with complete responses for the 38 items on outpatient experience of care in public hospitals were included in data analysis.

Of the 2605 participating inpatients, 2538 had no missing data in general characteristics and 2379 were 18 years old and above. We excluded two items that had high rates of “not applicable” responses (≧30%). We then excluded cases with responses “cannot tell” (*n*=160) and “not applicable” (*n*=709). Finally, 1510 cases with complete responses for the 40 items on inpatient experience of care in public hospitals were included in data analysis.

### Data analysis

#### Coding and scoring rules for patient experience items

There were five types of answering options for patient experience items. The coding rule for each type was shown in Table 1. Regarding the scoring rule, the percent of the highest code for each item was used. For example, if a participant chose a “negative” response (coded as 2) for a 1–5 Likert-type item (i.e. the highest code was 5), then for that participant the item was scored 40.0 (i.e. 2/5*100). The item on general satisfaction with hospital care also had the five-point Likert-type answering options, the score of which was calculated in the same way. After conducting the psychometric evaluation on the PREM-CCH, our research team grouped patient experience items concerning the same theme into the same Factor. The score for each Factor was calculated by taking the mean of the scores of the items under it. The total score for the PREM-CCH was calculated by taking the mean of the scores of all included items [32].

**Table 1:** Types and coding of answering options for the patient experience items

Type of answering options	Coding
1. The five-point Likert type	Very negative=1
	Negative=2
	Neither negative nor positive=3
	Positive=4
	Very positive=5
2. The three-category frequency type	Never=1
	Sometimes=2
	Often=3
3. The four-category frequency type	Never=1
	Sometimes=2
	Often=3
	Always=4
4. The yes/no type	No=1
	Yes=2
5. The yes/no and one additional response type	No=1
	I did not care/Not sure=2
	Yes=3

#### Measurement of psychometric properties

Separate psychometric evaluation was carried out on the outpatient PREM-CCH and the inpatient PREM-CCH, by conducting exploratory factor analysis and examining the internal consistency, construct validity and criterion validity. Two rounds of exploratory factor analysis and internal consistency assessment were performed for outpatient and inpatient PREM-CCHs respectively to identify Factors.

##### Exploratory factor analysis

To identify Factors, exploratory factor analysis was performed under the condition that the Kaiser-Meyer-Olkin (KMO) measure of sampling adequacy was > 0.6 and the Bartlett’s test of sphericity was statistically significant [33]. The principal components method was used to extract Factors, which was based on Eigenvalues > 1. Varimax was used to rotate Factors. Items were assigned to the Factor on which they had the highest loading. If the highest factor loading of an item was less than 0.3, it would not be assigned to any Factor [34].

##### Internal consistency

Internal consistency is an important psychometric property that examine to what extent multiple items measure the same underlying concept [35]. To assess internal consistency, Cronbach’s α for the whole instrument and for each Factor were calculated as well as the corrected item-total correlations and inter-item correlations in each Subscale. Internal consistency was justified if Cronbach’s α ≧0.7 and correlations ≧0.3 [36–38].

##### Construct validity

Construct validity is the extent to which the scores for a certain instrument are related to other measures that are expected to be correlated [35, 39]. In the present study, we hypothesized that scores for the PREM-CCH were correlated to the item on general satisfaction with hospital care. Two steps were taken to assess construct validity. First, Spearman’s rank correlation coefficients (rho) were used to investigate correlations between individual items of patient experience and the item on general satisfaction with hospital care. Second, multivariate linear regression was performed. The score for the item on general satisfaction with hospital care was treated as the dependent variable and the scores for Factors were treated as independent variables. Construct validity was justified if significant correlations were found [32].

##### Criterion validity

Criterion validity is the extent to which an instrument’s score is related to a gold standard [35]. While there is no gold standard for measures of patient experience, correlations between patient experience and patient satisfaction, assessed by Spearman’s rank correlation coefficients (rho), were employed in the present study to examine criterion validity. If moderate (rho 0.40–0.69) or strong (rho 0.70–0.89) correlations were found between the total score for the PREM and the score for the item on general satisfaction with hospital care, criterion validity was justified [36].

#### Statistical analysis

Descriptive statistics were performed to analyze the general characteristics of participants. The variables included were sex, age group (18–24 year, 25–34 years, 35–44 years, 45–54 years, 55–64 years, ≧65 years), educational level (primary school and below; middle school; high school; college and above), and self-reported disease seriousness (a 1–5 Likert scale: 1=not serious; 2=not very serious; 3=neither serious nor not serious; 4=rather serious; 5=very serious. Annual household income was divided into five groups: < 30,000 Chinese yuan; 30,000 to less than 80,000 Chinese yuan; 80,000 to less than 300,000 Chinese yuan; ≧300,000 Chinese yuan; cannot tell. As for the annual household income less than 1.0% cases had a response of “cannot tell”, we kept them in order to maximize the data available for psychometric evaluation. The *χ*^*2*^ test and *t* test were used to examine the differences in general characteristics between outpatients and inpatients. All analyses were performed in IBM SPSS 22.0 [40], using a 0.05 significance level.

## Results

### General characteristics

The general characteristics of participants were presented for outpatients and inpatients, respectively (Table 2). Over 60% outpatients were female while sex distribution among inpatients were nearly equal. Compared to outpatients, more inpatients were older than 55 years, less wealthy, had lower education level, and rated their diseases as serious.

**Table 2:** General characteristics of participants, by outpatients and inpatients

	Outpatients (***n***=2293)		Inpatients (***n***=1510)		***p*** value
	%	n	%	n	
**Age (Mean±SD)**	41.1±17.3		55.7±17.5		< 0.001
**Age group (years**)					< 0.001
18–24	17.4	398	4.2	64	
25–34	28.5	653	10.6	160	
35–44	14.7	337	10.7	161	
45–54	16.0	368	21.7	327	
55–64	10.6	242	18.5	279	
≧65	12.9	295	34.4	519	
**Sex**					< 0.001
Men	37.2	854	50.4	761	
Women	62.8	1439	49.6	749	
**Educational level**					< 0.001
Primary school and below	18.8	431	40.1	605	
Middle school	28.4	652	34.3	518	
High school	15.0	344	12.1	183	
College and above	37.8	866	13.5	204	
I**ncome group**					< 0.001
First group	38.7	887	55.0	831	
Second group	43.9	1007	33.2	502	
Third group	14.8	340	10.0	151	
Fourth group	1.5	35	1.0	15	
Cannot tell	1.0	24	0.7	11	
**Self-reported disease seriousness**					< 0.001
Not serious	21.8	500	7.9	119	
Not very serious	14.9	342	8.1	123	
Neither serious nor not serious	38.4	881	19.2	290	
Rather serious	21.9	502	44.7	675	
Very serious	3.0	68	20.1	303	

### Psychometric evaluation of the outpatient PREM-CCH

#### First round of exploratory factor analysis

The KMO value was 0.9 and the Bartlett’s test of sphericity was statistically significant. Eigenvalues ranged from 1.0 to 10.3. Factor loadings varied between 0.4 and 0.9. Nine Factors were extracted at this stage.

Factor 1 assessed three themes, i.e. *Communication and information*, *Professional competence*, *Environment and facilities*. Twelve items were assigned to this one Factor.

Factor 2 assessed *Communication and information*. Items assigned to this Factor were related to the content and effect of communication between medical professionals and patients as well as the attitudes of doctors during communication.

Factor 3 assessed *Medical costs*. Four items assessed how patients perceived the health care expenses on registry, diagnosis and treatment, medications, tests and examinations and the overall experience of medical expenses during this visit. One item assessed the appropriateness of the quantity of medications prescribed by doctors.

Factor 4 assessed *Efficiency*, which assessed patient experience of waiting time at different sections during this visit.

Factor 5 assessed *Communication and information* and *Professional competence*. Three items were assigned to this Factor. Two items assessed the responsiveness during the communication between doctors and patients. The other one assessed whether medical staff followed standards during the patient’s visit, i.e. checking the patient’s identity before conducting diagnosis and treatment.

Factor 6 assessed *Hospital recommendation*. One item evaluated patient willingness to revisit the same hospital and the other evaluated patient willingness to recommend this hospital to others.

Factor 7 assessed *Professional competence*. Two items assigned to this Factor assessed whether medical staff followed standards during the patient’s visit.

Factor 8 assessed *Communication and information*. One item was centered on patient involvement during the communication with doctors. The other two items were concerned with privacy protection of the patient during diagnosis and treatment.

Factor 9 assessed *Environment and facilities*. One item examined various ways of registration, and the other checked the channels of making complaints and suggestions to the hospital.

#### First round of internal consistency assessment

The Cronbach’s α of the whole outpatient PREM-CCH was 0.9, and the Cronbach’s alpha of different Factors ranged from 0.3 to 0.9. As the Cronbach’s α of Factors 7, 8 and 9 were < 0.7, we excluded those three Factors. For Factor 1, the inter-item correlations between Q41 and another four items were < 0.3, therefore we excluded Q41. The inter-item correlations between Q26, Q36 and Q40 were < 0.3, and among the item-total correlations of those three items, the one of Q26 was the smallest, hence we excluded Q26. The inter-item correlation between Q18 and Q36 was < 0.3, and the item-total correlation of Q18 was larger than that of Q36, so we excluded Q36. As a result, 29 items were included into the second round of exploratory factor analysis and internal consistency assessment.

#### Second round of exploratory factor analysis

The KMO value was 0.9 and the Bartlett’s test of sphericity was statistically significant. Eigenvalues ranged from 1.1 to 9.0. Factor loadings varied between 0.5 and 0.8. Six Factors were extracted at this stage.

Factor 1 contained 11 items with four items covering *Communication and information*, four items covering *Environment and facilities* and the other three covering *Professional competence*.

Factor 2 assessed *Medical costs*. Same items were assigned to this Factor as in the first round of exploratory factor analysis.

Factor 3 assessed *Communication and information*, which was relevant to the effect of communication between doctors and patients, e.g. whether doctors made concrete explanations and whether patients could understand their illness and treatment afterwards.

Factor 4 assessed *Efficiency*. Items assigned to this Factor were the same as those in the first round of exploratory factor analysis.

Factor 5 assessed *Communication and information* and *Professional competence*, which were the same as Factor 5 in the first round of exploratory factor analysis.

Factor 6 assessed *Hospital recommendation*. The two items assigned to this Factor were the same as those in the first round of exploratory factor analysis.

#### Second round of internal consistency assessment

The Cronbach’s α of the whole outpatient PREM-CCH was 0.9, and Cronbach’s α of the six Factors ranged from 0.7 to 0.9. As to Factor 1, the inter-item correlations between Q21 and another three items were < 0.3, so we excluded Q21. Although satisfactory internal consistency was achieved, it was difficult to interpret the results: Factors 1, 3 and 5 overlapped in themes, and Factors 1 and 5 contained multiple themes, respectively. Therefore, we grouped items of the same theme together, and reached the following five Factors and 22 items.

Factor 1 assessed *Communication and information*, including items Q13, Q14, Q15, Q16, Q17, Q18, Q19, Q22.

Factor 2 assessed *Professional competence*, including items Q28, Q32, Q34, Q40. The Cronbach’s α of this Factor was less than the threshold 0.7, but it could increase to 0.73 if we deleted Q28. Therefore, we excluded Q28.

Factor 3 assessed *Medical costs*, including items Q46, Q47, Q48, Q49, Q51.

Factor 4 assessed *Efficiency*, including items Q42, Q43, Q44, Q45.

Factor 5 assessed *Hospital recommendation*, including items Q54 and Q55.

As to the Factor assessing *Environmental and facilities*, its Cronbach’s α was 0.68 that could not increase to 0.7 no matter which item of this Factor was deleted. Hence, we excluded this Factor.

Cronbach’s α of the revised outpatient PREM-CCH was 0.9. Cronbach’s α of the five Factors ranged from 0.7 to 0.9 (Table 3). The inter-item correlations were all > 0.3, ranging from 0.3 to 0.7; the corrected item-total correlations between individual items and their assigned Factors were all > 0.3, ranging from 0.5 to 0.7.

**Table 3:** Factor Cronbach’s α and Spearman’s rank correlation coefficients (rho) between individual patient experience items and the item on general satisfaction with hospital care in the revised outpatient PREM-CCH (n=2293)

Factors and items	Cronbach’s α and rho
**Factor 1** ***Communication and information*** **(8 items)**	**0.9**^a^
Q13. Did the doctor explain your illness and related issues concretely?	0.4
Q14. Do you understand your condition clearly after this visit?	0.3
Q15. Do you understand the treatment protocol of your illness clearly after the visit?	0.3
Q16. Could you get timely responses when you asked questions?	0.3
Q17. Could you get timely help when you needed?	0.3
Q18. Were medical professionals friendly and respectful during this visit?	0.5
Q19. Did the doctor listen to the description of your condition patiently during this visit?	0.5
Q22. Are you satisfied with the communication between you and the medical professionals during this visit?	0.5
**Factor 2** ***Professional competence*** **(3 items)**	**0.7**^a^
Q32. Do you think the medical professionals have done sufficient inquiry and medical check-up?	0.5
Q34. Do you think the medical professionals followed the standard procedures during this visit?	0.5
Q40. What do you think of the skills of the medical professionals in this hospital?	0.5
**Factor 3** ***Medical costs*** **(5 items)**	**0.8**^a^
Q46. Do you think the amount of money you spent this time was worthwhile regarding to your condition?	0.5
Q47. Do you think the amount of money you spent on registration, diagnosis and treatment this time was reasonable?	0.4
Q48. Do you think the amount of money you spent on medications this time was reasonable?	0.4
Q49. Do you think the amount of money you spent on tests and examinations this time was reasonable?	0.4
Q51. Do you think the medications the doctor prescribed this time were reasonable?	0.4
**Factor 4** ***Efficiency*** **(4 items)**	**0.8**^a^
Q42. Do you think the waiting time for registry and paying fees was reasonable?	0.3
Q43. Do you think the waiting time for seeing the doctor was reasonable?	0.3
Q44. Do you think the waiting time for examinations was reasonable?	0.3
Q45. Do you think the waiting time for getting medications was reasonable?	0.3
**Factor 5** ***Hospital recommendation*** **(2 items)**	**0.8**^a^
Q54. Would you still go to this hospital if you need to see the doctor next time?	0.4
Q55. Would you recommend this hospital to your relatives and friends if they are sick?	0.4

^a^: Factor Cronbach’s α. All Spearman’s rank correlation coefficients (rho) were significant at the 0.05 level

#### Construct validity

As shown in Table 3, all individual patient experience items in the revised outpatient PREM-CCH had statistically significant correlations with the item on general satisfaction with hospital care. In the multivariate regression analysis (Table 4), all Factors were statistically significantly associated with the item on general satisfaction with hospital care.

**Table 4:** Multivariate linear regression on general satisfaction with hospital care among outpatients, by five Factors (n=2293)

	***Standardized coefficients***	***p*** values
Factor 1 *Communication and information*	0.2	< 0.001
Factor 2 *Professional competence*	0.3	< 0.001
Factor 3 *Medical costs*	0.2	< 0.001
Factor 4 *Efficiency*	0.1	< 0.001
Factor 5 *Hospital recommendation*	0.2	< 0.001
Adjusted R^2^	0.5	

#### Criterion validity

Criterion validity was supported for the revised outpatient PREM-CCH, as the total score for the instrument correlated moderately with the score for the item on general satisfaction with hospital care (rho=0.65). The final validated version of the outpatient PREM-CCH is presented in Additional file 1: Table A1.

### Psychometric evaluation of the inpatient PREM-CCH

#### First round of exploratory factor analysis

The KMO value was 0.9 and the Bartlett’s test of sphericity was statistically significant. Eigenvalues ranged from 1.1 to 10.1. Factor loadings varied between 0.4 and 0.9. Nine factors were extracted at this stage.

Factor 1 assessed three themes, i.e. *Communication and information*, *Professional competence*, *Environment and facilities*. Twelve items were assigned to this factor.

Factor 2 assessed *Medical costs*. Four items assessed how patients perceived the health care expenses on registry, diagnosis and treatment, medications, tests and examinations and the overall experience of medical expenses during this visit. One item assessed self-perceived appropriateness of medications prescribed by doctors.

Factor 3 assessed *Efficiency*. Three items assessed patient experience of waiting time at different sections during this visit, including the waiting time for registration and paying fees, for seeing the doctor, and for examinations.

Factor 4 assessed *Communication and information*. Three items assigned to this factor were related to the content and effect of communication between medical professionals and patients.

Factor 5 assessed three themes, with two items under *Health outcomes*, one under *Professional competence*, and another one under *Environment and facilities*.

Factor 6 assessed *Communication and information* and *Professional competence*. Two items assessed the responsiveness of medical professionals, and one item assessed whether medical professionals complied with diagnosis regulations during the patient’s visit.

Factor 7 assessed *Communication and information*, and *Environment and facilities*. Two items were concerned with privacy protection of the patient during diagnosis and treatment. One item was related to multiple ways of registration, and the other was about channels to make complaints and suggestions to the hospital.

Factor 8 assessed *Hospital recommendation*. One item assessed the patient’s willingness to revisit the same hospital and the other assessed the patient’s willingness to recommend this hospital to others.

Factor 9 assessed three subscales, i.e. *Communication and information*, *Professional competence*, and *Medical costs*. One item examined patient engagement into decision making concerning their treatment protocols. Two items examined whether medical professionals complied with professional procedures during treatment, e.g. informing patients of potential risks, preventative methods and follow-ups. The other one item examined the necessity of the tests and examinations taken during this visit.

#### First round of internal consistency

The Cronbach’s α of the whole inpatient PREM-CCH was 0.9. The Cronbach’s α of the nine Factors ranged from 0.3 to 0.9. We excluded Factors 7 and 9 as the Cronbach’s α of neither Factor was above 0.7, and even if we excluded individual items, the Cronbach’s α was still < 0.7. As to Factor 1, we excluded items Q21, Q26, Q27, Q31 and Q36, between which and the other items under Factor 1, the inter-item correlations were < 0.3. With regard to Factor 2, we excluded Q28, as the Cronbach’s α of Factor 2 (0.68) could increase to 0.77 if Q28 was deleted. A total of 26 items were included into the second round of exploratory factor analysis and internal consistency assessment.

#### Second round of exploratory factor analysis

The KMO value was 0.9 and the Bartlett’s test of sphericity was statistically significant. Eigenvalues ranged from 1.0 to 8.1. Factor loadings varied between 0.5 and 0.9. Seven Factors were extracted at this stage.

Factor 1 contained 7 items with three items covering *Communication and information*, two items concerning *Environment and facilities*, and the other two items related to *Professional competence*.

Factor 2 and Factor 3 assessed *Medical costs* and *Efficiency*, respectively. Same items were assigned to these two Factors as in the first round of exploratory factor analysis.

Factor 4, as Factor 5 in the first round of exploratory factor analysis, assessed three themes, with two items under *Health outcomes*, one under *Professional competence*, and another one under *Environment and facilities*.

Factor 5, as Factor 4 in the first round of exploratory factor analysis, assessed *Communication and information*.

Factor 6 assessed *Communication and information*, under which the two items were related to the responsiveness of medical professionals.

Factor 7, as Factor 8 in the first round of exploratory factor analysis, assessed *Hospital recommendation*.

#### Second round of internal consistency

The Cronbach’s α of the whole inpatient PREM-CCH was 0.9, and Cronbach’s α of the seven Factors ranged from 0.7 to 0.8. As there were Factors that overlapped in themes, we grouped items containing the same theme together, and reached the following six Factors and 19 items.

Factor 1 assessed *Communication and information*, including items Q13, Q14, Q15, Q16, Q17, Q18, Q19, Q22. As the inter-item correlations between Q14, Q15, Q16, Q17 and the other items were < 0.3, we excluded those four items.

Factor 2 assessed *Professional competence*, including items Q32, Q34, Q40.

Factor 3 assessed *Medical costs*, including items Q46, Q47, Q48, Q49, Q51.

Factor 4 assessed *Efficiency*, including items Q42, Q43, Q44.

Factor 5 assessed *Health outcomes*, including items Q16 and Q17.

Factor 6 assessed *Hospital recommendation*, including items Q54 and Q55.

Regarding the Factor assessing *Environmental and facilities*, its Cronbach’s α was less than 0.7; we, therefore, excluded that Factor.

The Cronbach’s α of the revised inpatient PREM-CCH was 0.9, which representing good internal consistency. Cronbach’s α of the six Factors ranged from 0.7 to 0.8 (Table 5). The corrected item-total correlations between individual items and their assigned Factors were all > 0.3, ranging from 0.5 to 0.8; the inter-item correlations were all > 0.3, ranging from 0.4 to 0.7.

**Table 5:** Factor Cronbach’s α and Spearman’s rank correlation coefficients (rho) between individual patient experience items and the item on general satisfaction with hospital care in the revised inpatient PREM-CCH (n=1510)

Factors and items	Cronbach’s α and rho
**Factor 1** ***Communication and information*** **(4 items)**	**0.8**^a^
Q13. Did the doctor explain your illness and related issues concretely?	0.4
Q18. Were medical professionals friendly and respectful during this visit?	0.5
Q19. Did the doctor listen to the description of your condition patiently during this visit?	0.4
Q22. Are you satisfied with the communication between you and the medical professionals during this visit?	0.5
**Factor 2** ***Professional competence*** **(3 items)**	**0.7**^a^
Q32. Do you think the medical professionals have done sufficient inquiry and medical check-up?	0.4
Q34. Do you think the medical professionals followed the standard procedures during this visit?	0.5
Q40. What do you think of the skills of the medical professionals in this hospital?	0.5
**Factor 3** ***Medical costs*** **(5 items)**	**0.8**^a^
Q46. Do you think the amount of money you spend this time was worthwhile regarding to your condition?	0.4
Q47. Do you think the amount of money you spend on registration, diagnosis and treatment this time was reasonable?	0.4
Q48. Do you think the amount of money you spend on medications this time was reasonable?	0.4
Q49. Do you think the amount of money you spend on tests and examinations this time was reasonable?	0.4
Q51. Do you think the medications the doctor prescribed this time were reasonable?	0.4
**Factor 4** ***Efficiency*** **(3 items)**	**0.8**^a^
Q42. Do you think the waiting time for registration and paying fees was reasonable?	0.3
Q43. Do you think the waiting time for seeing the doctor was reasonable?	0.3
Q44. Do you think the waiting time for examinations was reasonable?	0.3
**Factor 5** ***Health outcomes*** **(2 items)**	**0.8**^a^
Q38. Could the treatment relief your symptoms and pain effectively?	0.4
Q39. Compare to your expectation, how do you evaluate the treatment outcome so far?	0.4
**Factor 6** ***Hospital recommendation*** **(2 items)**	**0.7**^a^
Q54. Would you still go to this hospital if you need to see the doctor next time?	0.3
Q55. Would you recommend this hospital to your relatives and friends if they are sick?	0.3

^a^: Factor Cronbach’s α. All Spearman’s rank correlation coefficients (rho) were significant at the 0.05 level

#### Construct validity

As shown in Table 5, all individual items in the revised inpatient PREM-CCH had statistically significant correlations with the item on general satisfaction with hospital care. In the multivariate regression analysis (Table 6), all Factors were statistically significantly associated with the items on general satisfaction with hospital care.

**Table 6:** Multivariate linear regression on general satisfaction with hospital care among inpatients, by six Factors (n=1510)

	***Standardized coefficients***	***p*** values
Factor 1 *Communication and information*	0.2	< 0.001
Factor 2 *Professional competence*	0.2	< 0.001
Factor 3 *Medical costs*	0.2	< 0.001
Factor 4 *Efficiency*	0.1	< 0.001
Factor 5 *Health outcomes*	0.1	0.001
Factor 6 *Hospital recommendation*	0.2	< 0.001
Adjusted R^2^	0.5	

#### Criterion validity

Criterion validity was supported for the revised inpatient PREM-CCH, as the total score for the instrument correlated moderately with the score for the item on general satisfaction with hospital care (rho=0.63). The final validated version of the inpatient PREM-CCH is presented in Additional file 1: Table A2.

## Discussion

This present study was conducted to validate the previously developed PREM-CCH for capturing patient experience of care in Chinese public hospitals [29]. The PREM-CCH was tested among 2293 outpatients and 1510 inpatients in the context of Chinese public hospitals and its psychometric properties were evaluated in the present study. The PREM-CCH demonstrated satisfactory internal consistency. Construct validity and criterion validity were also verified. The validated final version of the outpatient PREM-CCH contained 22 items and five Factors, i.e. *Communication and information*, *Professional competence*, *Medical costs*, *Efficiency*, and *Hospital recommendation*. The validated final version of the inpatient PREM-CCH contained 19 items and six Factors, i.e. *Communication and information*, *Professional competence*, *Medical costs*, *Efficiency*, *Health outcomes*, and *Hospital recommendation*.

To the best of our knowledge, this patient experience survey instrument is one of the first validated PREMs capturing patient experience of care provided in Chinese public hospitals. Available validated instruments focus more on inpatient care [41–43]. The outpatient and inpatient population who will use this PREM-CCH have been involved in its development, as patient experience items have been derived from the patient perspective. Data from both urban and rural areas have been included to conduct psychometric evaluation. As a result, this validated PREM-CCH consists of a basic set of items important to outpatients and inpatients that could be applicable to public hospitals in China and actionable to inform quality improvement initiatives.

Both outpatient and inpatient PREM-CCHs validated in the present study are based on the same 40 patient experience items except that two items on *Health outcomes* are designed exclusively for inpatients. It should be difficult to assess the effect of care provided at the end of an outpatient visit, as no regimen have been implemented yet. However, a patient might have a preliminary assessment on the experience of *Health outcomes* on the day of discharge. In the inpatient PREM-CCH, the Factor of *Health outcomes* has been justified.

As to the Factor of *Communication and information*, both outpatient and inpatient PREM-CCHs contain the same items on attitudes of medical professionals when communicating with patients and an item relevant to the overall assessment of communication, which have also been addressed in other validated patient experience instruments [44, 45]. In the present study, compared to the inpatient PREM-CCH, the outpatient PREM-CCH covered four more items on the impact of communication and the responsiveness of hospital staff. As for outpatients, the encounter with medical professionals is substantially shorter compared to inpatients, it should be of critical importance to outpatients that medical professional can help them understand their illness and relevant medical treatment during the visit. Therefore, timely communication and sufficient information are valued more by outpatients and the corresponding patient experience items are included in the outpatient PREM-CCH.

Regarding the Factor of *Efficiency*, waiting time for various sections are assessed in both outpatient and inpatient PREM-CCHs, covering registry, paying fees, seeing the doctor and taking examinations. However, in the outpatient PREM-CCH another item on *Efficiency* is included, i.e. waiting time for getting medications. In most cases, outpatients need to go to the drug dispensing area in a Chinese public hospital and get drugs themselves during the visit, while inpatients could have their drugs in the ward administered directly by nurses. This situation has been observed in the present study and manifested in the Factor of *Efficiency*.

Items within the Factors of *Professional competence*, *Medical costs* and *Hospital recommendation* are the same for both outpatient and inpatient PREM-CCHs. With respect to *Professional competence*, items included not only reflect medical professionals’ skills but also the necessary and standard procedures that medical professionals should follow. With regard to *Medical costs*, patient experience on the expenses for multiple items are measured, including registry, diagnosis and treatment, medications, tests and examinations. As to *Hospital recommendation*, both patients’ willingness to continue visiting the same hospital and their intention to recommend the hospital are measured.

The present study has several limitations. First, the study design might have been stronger if testing of reproductivity could be performed to examine the extent to which repeated measurements provide similar answers. Test-retest reliability is needed in further studies. Second, although the number of response types were reduced from five to three in the validated PREM-CCH, it might widen the gap between answering options if targeted users conduct coding and scoring of the instrument. On one hand, different items for outpatients and inpatients were identified in this validated PREM-CCH, which means that this instrument could differentiate key aspects between outpatient and inpatient experience and could be used in the performance evaluation of Chinese public hospitals for quality improvement. On the other hand, further studies should be carried out by using a modified version of this PREM-CCH where a uniform response format is applied. Third, the generalization of the findings of this study might be limited, as public hospitals in only four Chinese Provinces were included. However, the four sampled Provinces are all listed among the national pilots of the health-care system reform. The sampled hospitals cover both urban and rural areas in the eastern, middle and western parts of China. The findings of this study could be set as examples for the other regions in China. Finally, PROMs are not included into the present study. As patient experience of hospital care is linked to health, it would be good to use this PREM-CCH in conjunction with validated PROMs to better inform hospital managers and health authorities on how to achieve quality improvement.

## Conclusions

To the best of our knowledge, this PREM-CCH is one of the first validated instruments to measure patient experience of care in the context of Chinese public hospitals. Factors that matter to outpatients and inpatients have been identified, respectively, and constitute an outpatient PREM-CCH and an inpatient PREM-CCH of satisfactory internal consistency, construct validity and criterion validity. Further studies should be carried out to link PROMs to this PREM-CCH and to explore how to use this instrument to support quality improvement in Chinese public hospitals.

## Supplementary Information

**Additional file 1: Table A1**. The final validated version of the outpatient PREM-CCH. **Table A2**. The final validated version of the inpatient PREM-CCH.

## Data Availability

The data generated during this study are not publicly available, due to the reason that sufficient information is contained to enable readers to identify sampled hospitals, but a de-identified analytical file is available from the corresponding author on reasonable request.

## Abbreviations

PREM-CCH: Patient-Reported Experience Measure for Care in Chinese Hospitals
PREMs: Patient-Reported Experience Measures
PROMs: Patient-Reported Outcome Measures
NHFPC: National Health and Family Planning Commission
