# Supplementary material for: Validation of the patient-reported experience measure for care in Chinese hospitals (PREM-CCH)
Source: Int J Equity Health. 2021 Jan 7;20:25. doi: 10.1186/s12939-020-01370-6 (PMC7791723; doi:10.1186/s12939-020-01370-6)
Supplement: Supplementary file 1 — Additional file 1: Table A1. The final validated version of the outpatient PREM-CCH. Table A2. The final validated version of the inpatient PREM-CCH. [file 12939_2020_1370_MOESM1_ESM.docx]

## Appendix

**Table A1. The final validated version of the outpatient PREM-CCH**

| **COMMUNICATION AND INFORMATION** |
| --- |
| 1. **Did the doctor explain your illness and related issues concretely?**   ^1^**□** Not concretely at all  ^2^**□** Not very concretely  ^3^**□** Neither concretely nor not concretely  ^4^**□** Concretely  ^5^**□** Very concretely |
| 1. **Do you understand your condition clearly after this visit?**   ^1^**□** Not clearly at all  ^2^**□** Not very clearly  ^3^**□** Neither clearly nor not clearly  ^4^**□** Clearly  ^5^**□** Very clearly |
| 1. **Do you understand the treatment protocol of your illness clearly after this visit?**   ^1^**□** Not clearly at all  ^2^**□** Not very clearly  ^3^**□** Neither clearly nor not clearly  ^4^**□** Clearly  ^5^**□** Very clearly |
| 1. **Could you get timely responses when you asked questions?**   ^1^**□** Never  ^2^**□** Sometimes  ^3^**□** Often |
| 1. **Could you get timely help when you needed?**   ^1^**□** Never  ^2^**□** Sometimes  ^3^**□** Often |
| 1. **Were the medical professionals friendly and respectful during this visit?**   ^1^**□** Not friendly or respectful at all  ^2^**□** Not very friendly or respectful  ^3^**□** Moderate  ^4^**□** Friendly and respectful  ^5^**□** Very friendly and respectful |
| 1. **Did the doctor listen to the description of your condition patiently during this visit?**   ^1^**□** Not patiently at all  ^2^**□** Not very patiently  ^3^**□** Neither patiently nor not patiently  ^4^**□** Patiently  ^5^**□** Very patiently |
| 1. **Are you satisfied with the communication between you and the medical professionals during this visit?**   ^1^**□** Not satisfied at all  ^2^**□** Not satisfied  ^3^**□** Neither satisfied nor not satisfied  ^4^**□** Satisfied  ^5^**□** Very satisfied |
| **PROFESSIONAL COMPETENCE** |
| 1. **Do you think the medical professionals have done sufficient inquiry and medical check-up?**   ^1^**□** Not sufficient at all  ^2^**□** Not very sufficient  ^3^**□** Neither sufficient nor not sufficient  ^4^**□** Sufficient  ^5^**□** Very sufficient |
| 1. **Do you think the medical professionals followed the standard procedures during this visit?**   ^1^**□** Not standard at all  ^2^**□** Not very standard  ^3^**□** Neither standard nor not standard  ^4^**□** Standard  ^5^**□** Very standard |
| 1. **What do you think of the skills of the medical professionals in this hospital?**   ^1^**□** Not high at all  ^2^**□** Not very high  ^3^**□** Moderate  ^4^**□** High  ^5^**□** Very high |
| **MEDICAL COSTS** |
| 1. **Do you think the amount of money you spent this time was worthwhile regarding to your condition?**   ^1^**□** Not worthwhile at all  ^2^**□** Not very worthwhile  ^3^**□** Neither worthwhile nor not worthwhile  ^4^**□** Worthwhile  ^5^**□** Very worthwhile |
| 1. **Do you think the amount of money you spent on registration, diagnosis and treatment this time was reasonable?**   ^1^**□** Not reasonable at all  ^2^**□** Not very reasonable  ^3^**□** Neither reasonable nor not reasonable  ^4^**□** Reasonable  ^5^**□** Very reasonable |
| 1. **Do you think the amount of money you spent on medications this time was reasonable?**   ^1^**□** Not reasonable at all  ^2^**□** Not very reasonable  ^3^**□** Neither reasonable nor not reasonable  ^4^**□** Reasonable  ^5^**□** Very reasonable |
| 1. **Do you think the amount of money you spent on tests and examinations this time was reasonable?**   ^1^**□** Not reasonable at all  ^2^**□** Not very reasonable  ^3^**□** Neither reasonable nor not reasonable  ^4^**□** Reasonable  ^5^**□** Very reasonable |
| 1. **Do you think the medications the doctor prescribed this time were reasonable?**   ^1^**□** Not reasonable at all  ^2^**□** Not very reasonable  ^3^**□** Neither reasonable nor not reasonable  ^4^**□** Reasonable  ^5^**□** Very reasonable |
| **EFFICIENCY** |
| 1. **Do you think the waiting time for registration and paying fees was reasonable?**   ^1^**□** Very long  ^2^**□** A little bit long  ^3^**□** Neither short nor long  ^4^**□** Relatively short  ^5^**□** Very short |
| 1. **Do you think the waiting time for seeing the doctor was reasonable?**   ^1^**□** Very long  ^2^**□** A little bit long  ^3^**□** Neither short nor long  ^4^**□** Relatively short  ^5^**□** Very short |
| 1. **Do you think the waiting time for examinations was reasonable?**   ^1^**□** Very long  ^2^**□** A little bit long  ^3^**□** Neither short nor long  ^4^**□** Relatively short  ^5^**□** Very short |
| 1. **Do you think the waiting time for getting medications was reasonable?**   ^1^**□** Very long  ^2^**□** A little bit long  ^3^**□** Neither short nor long  ^4^**□** Relatively short  ^5^**□** Very short |
| **HOSPITAL RECOMMENDATION** |
| 1. **Would you still go to this hospital if you need to see the doctor next time?**   ^1^**□** No  ^2^**□** Not sure  ^3^**□** Yes |
| 1. **Would you recommend this hospital to your relatives and friends if they are sick?**   ^1^**□** No  ^2^**□** Not sure  ^3^**□** Yes |
| **GENERAL SATISFACTION** |
| 1. **In general, are you satisfied with the services in this hospital?**   ^1^**□** Not satisfied at all  ^2^**□** Not satisfied  ^3^**□** Neither satisfied nor not satisfied  ^4^**□** Satisfied  ^5^**□** Very satisfied |

**Table A2. The final validated version of the inpatient PREM-CCH**

| **COMMUNICATION AND INFORMATION** |
| --- |
| 1. **Did the doctor explain your illness and related issues concretely?**   ^1^**□** Not concretely at all  ^2^**□** Not very concretely  ^3^**□** Neither concretely nor not concretely  ^4^**□** Concretely  ^5^**□** Very concretely |
| 1. **Were the medical professionals friendly and respectful during this visit?**   ^1^**□** Not friendly or respectful at all  ^2^**□** Not very friendly or respectful  ^3^**□** Moderate  ^4^**□** Friendly and respectful  ^5^**□** Very friendly and respectful |
| 1. **Did the doctor listen to the description of your condition patiently during this visit?**   ^1^**□** Not patiently at all  ^2^**□** Not very patiently  ^3^**□** Neither patiently nor not patiently  ^4^**□** Patiently  ^5^**□** Very patiently |
| 1. **Are you satisfied with the communication between you and the medical professionals during this visit?**   ^1^**□** Not satisfied at all  ^2^**□** Not satisfied  ^3^**□** Neither satisfied nor not satisfied  ^4^**□** Satisfied  ^5^**□** Very satisfied |
| **PROFESSIONAL COMPETENCE** |
| 1. **Do you think the medical professionals have done sufficient inquiry and medical check-up?**   ^1^**□** Not sufficient at all  ^2^**□** Not very sufficient  ^3^**□** Neither sufficient nor not sufficient  ^4^**□** Sufficient  ^5^**□** Very sufficient |
| 1. **Do you think the medical professionals followed the standard procedures during this visit?**   ^1^**□** Not standard at all  ^2^**□** Not very standard  ^3^**□** Neither standard nor not standard  ^4^**□** Standard  ^5^**□** Very standard |
| 1. **What do you think of the skills of the medical professionals in this hospital?**   ^1^**□** Not high at all  ^2^**□** Not very high  ^3^**□** Moderate  ^4^**□** High  ^5^**□** Very high |
| **MEDICAL COSTS** |
| 1. **Do you think the amount of money you spent this time was worthwhile regarding to your condition?**   ^1^**□** Not worthwhile at all  ^2^**□** Not very worthwhile  ^3^**□** Neither worthwhile nor not worthwhile  ^4^**□** Worthwhile  ^5^**□** Very worthwhile |
| 1. **Do you think the amount of money you spent on registration, diagnosis and treatment this time was reasonable?**   ^1^**□** Not reasonable at all  ^2^**□** Not very reasonable  ^3^**□** Neither reasonable nor not reasonable  ^4^**□** Reasonable  ^5^**□** Very reasonable |
| 1. **Do you think the amount of money you spent on medications this time was reasonable?**   ^1^**□** Not reasonable at all  ^2^**□** Not very reasonable  ^3^**□** Neither reasonable nor not reasonable  ^4^**□** Reasonable  ^5^**□** Very reasonable |
| 1. **Do you think the amount of money you spent on tests and examinations this time was reasonable?**   ^1^**□** Not reasonable at all  ^2^**□** Not very reasonable  ^3^**□** Neither reasonable nor not reasonable  ^4^**□** Reasonable  ^5^**□** Very reasonable |
| 1. **Do you think the medications the doctor prescribed this time were reasonable?**   ^1^**□** Not reasonable at all  ^2^**□** Not very reasonable  ^3^**□** Neither reasonable nor not reasonable  ^4^**□** Reasonable  ^5^**□** Very reasonable |
| **EFFICIENCY** |
| 1. **Do you think the waiting time for registration and paying fees was reasonable?**   ^1^**□** Very long  ^2^**□** A little bit long  ^3^**□** Neither short nor long  ^4^**□** Relatively short  ^5^**□** Very short |
| 1. **Do you think the waiting time for seeing the doctor was reasonable?**   ^1^**□** Very long  ^2^**□** A little bit long  ^3^**□** Neither short nor long  ^4^**□** Relatively short  ^5^**□** Very short |
| 1. **Do you think the waiting time for examinations was reasonable?**   ^1^**□** Very long  ^2^**□** A little bit long  ^3^**□** Neither short nor long  ^4^**□** Relatively short  ^5^**□** Very short |
| **HEALTH OUTCOMES** |
| 1. **Could the treatment relief your symptoms and pain effectively?**   ^1^**□** Not effectively at all  ^2^**□** Not very effectively  ^3^**□** Neither effectively nor not effectively  ^4^**□** Effectively  ^5^**□** Very effectively |
| 1. **Compare to your expectation, how do you evaluate the treatment outcome so far?**   ^1^**□** Not good at all  ^2^**□** Not very good  ^3^**□** Moderate  ^4^**□** Good  ^5^**□** Very good |
| **HOSPITAL RECOMMENDATION** |
| 1. **Would you still go to this hospital if you need to see the doctor next time?**   ^1^**□** No  ^2^**□** Not sure  ^3^**□** Yes |
| 1. **Would you recommend this hospital to your relatives and friends if they are sick?**   ^1^**□** No  ^2^**□** Not sure  ^3^**□** Yes |
| **GENERAL SATISFACTION** |
| 1. **In general, are you satisfied with the services in this hospital?**   ^1^**□** Not satisfied at all  ^2^**□** Not satisfied  ^3^**□** Neither satisfied nor not satisfied  ^4^**□** Satisfied  ^5^**□** Very satisfied |
